# Quantification of Organic Carbon Sequestered by Biogenic Iron Sulfide Minerals in Long-Term Anoxic Laboratory Incubations

**DOI:** 10.3389/fmicb.2022.662219

**Published:** 2022-04-27

**Authors:** Nader Nabeh, Cheyenne Brokaw, Aude Picard

**Affiliations:** School of Life Sciences, University of Nevada, Las Vegas, Las Vegas, NV, United States

**Keywords:** iron sulfide minerals, biomineral, microbial sulfate reduction, *Desulfovibrio*, mackinawite, greigite, organic carbon preservation

## Abstract

Organic carbon sequestration in sedimentary environments controls oxygen and carbon dioxide concentrations in the atmosphere. While minerals play an important role in the preservation of organic carbon, there is a lack of understanding about the formation and stability of organo-mineral interactions in anoxic environments, especially those involving authigenic iron sulfide minerals. In this study, we quantified organic carbon and nitrogen sequestered in biogenic iron sulfide minerals co-precipitated with sulfate-reducing bacteria (SRB) in freshwater and marine conditions in long-term laboratory experiments. The amounts of C and N associated with biogenic iron sulfide minerals increased with increasing cell biomass concentrations available in the media. C and N levels stabilized over the first 2 months of incubation and remained stable for up to 1 year. Crystalline mackinawite (FeS) formed in all experimental conditions and transformed to greigite only in some experimental conditions. We did not find evidence that this mineral transformation affected C and N levels, neither could we identify the factors that controlled greigite formation. Pyrite did not form in our experimental conditions. While C concentrations in minerals correlated with concentrations of reduced sulfate in both the freshwater and marine media, removal of OC by iron sulfide minerals was more efficient in freshwater than marine conditions. Removal of OC by iron sulfide minerals was also more efficient when cells were present (SRB biomass) in comparison with abiotic incubations with organic mixtures (e.g., tryptone, yeast extract, and casamino acids). Our study highlights the potential for biogenic iron sulfide minerals to quantitatively contribute to organic carbon preservation in anoxic environments.

## Introduction

The long-term preservation of organic carbon has significant impacts on the global carbon cycle. Over geological timescales, organic carbon sequestration removes carbon dioxide from the atmosphere and releases oxygen into the Earth’s atmosphere (Berner and Canfield, 1989; Berner, 1990; Keil, 2017; LaRowe et al., 2020). Sedimentary environments represent the largest reservoir of organic carbon. Anoxic and reduced conditions promote increased organic carbon burial (Jenkyns, 2010; Raven et al., 2019). Indeed, organic carbon deposition is typically enhanced in coastal and shelf environments, below zones of high primary productivity where oxygen-deficient zones can develop (Keil, 2017; Raven et al., 2021b). Anthropogenically driven climate change is promoting an expansion of oxygen-free zones in the ocean, leading to the enhanced delivery of OC to the seafloor and potentially enhanced preservation (Keil, 2017). While anoxic marine sediments represent the largest long-term organic carbon repository (Canfield, 1994; Burdige, 2007), freshwater sedimentary environments are also significant for the preservation of OC due to their sensitivity to changes in the carbon cycle, especially those driven by human activity (Boye et al., 2017). Microbial communities play an important role in the transformations of organic carbon (Singh et al., 2010; Cavicchioli et al., 2019; LaRowe et al., 2020). While microorganisms are typically assumed to degrade most organic carbon in the environment, they can also play a role in its preservation through mechanisms such as polymerization, sulfurization, or biomineralization (Ogawa et al., 2001; Ingalls et al., 2003, 2004; Chan et al., 2009; Jiao et al., 2010; Picard et al., 2019; Raven et al., 2021b).

Interactions between organic carbon and minerals play a role in long-term organic carbon (OC) preservation (Keil and Mayer, 2014; Hemingway et al., 2019; Kleber et al., 2021). The association of organic compounds with minerals is driven by multiple physical and chemical processes, including physical adsorption and/or strong bonding at mineral surfaces, insertion into clay interlayers, formation of mineral-OC aggregates, co-precipitation with authigenic minerals, incorporation into exopolymeric substances (EPS), and production of biogenic minerals by controlled mineralization (LaRowe et al., 2020). Ligand exchange, ion exchange, and hydrogen bonding are potential driving forces for the aggregation of organic matter and minerals that may strengthen organo-mineral interactions and thus provide a mechanism for OC preservation (Keil and Mayer, 2014). Minerals could stabilize and protect organic matter against alteration, such as weathering, decomposition, and microbial degradation, therefore preserving organic carbon derived from living and dead microbial biomass for long periods of time (Keil and Mayer, 2014).

In anoxic environments, the biogeochemical cycles of iron, carbon, and sulfur are intertwined through microbial activity (Rickard, 2012a; Jørgensen et al., 2019). Reduced sulfur produced by microbial sulfate reduction interacts with Fe(II) to form iron sulfide minerals but also interacts with Fe(III) minerals to form Fe(II) and intermediate-redox sulfur species (Rickard, 2012a; Picard et al., 2016). Sulfate-reducing microorganisms (SRM) can be found in a variety of aquatic environments and are responsible for 97% of Earth’s sulfide production in low-temperature environments (Rickard, 2012a). Iron-rich substrates seem to prevent the degradation of organic compounds by microbial metabolic activity. For example, organic carbon preservation occurs due to co-precipitation with ligand-Fe and with Fe oxides in a variety of environments, including surface marine and estuarine environments, as well as in hydrothermal plumes (Lalonde et al., 2012; Barber et al., 2017; Hoffman et al., 2018). The role of OC-Fe ligand and OC-Fe minerals in preserving OC in anoxic environments has hardly been studied (Barber et al., 2017). Sulfide produced by SRM promotes the sulfurization of organic carbon in a variety of marine environments (Raven et al., 2016a,b, 2021b). Additionally, through their influence on the formation of iron sulfide minerals, SRM could also play an important role in the long-term preservation of organic carbon in anoxic sedimentary environments. In laboratory experiments, SRM influence the physical and chemical properties of iron sulfide minerals, and also potentially their mineralogy (Picard et al., 2016, 2018; Mansor et al., 2019). Whether iron sulfide minerals associate tightly with cells (through mineral encrustation) or precipitate away from cells, they associate strongly with organic carbon derived from microbial biomass. Notably, iron sulfide minerals have a strong affinity for proteinaceous material (Picard et al., 2019). Microbial organic carbon associated with biogenic iron sulfide minerals precipitated in anoxic laboratory conditions is still detectable by *X*-ray microscopy after incubations of 2 years (Picard et al., 2019, 2021). Moreover, biogenic minerals are more stable than abiotic minerals precipitated with organic molecules, which indicate the importance of microbial biomass and cellular structures in the preservation of organic carbon.

Here, we quantified concentrations of organic carbon and nitrogen that biogenic iron sulfide minerals can sequester in long-term laboratory experiments. Biogenic minerals were precipitated in the presence of a marine SRB, *Desulfovibrio hydrothermalis* AM13, and a freshwater SRB, *Desulfovibrio magneticus* RS-1 and incubated for up to 1 year at their optimal growth temperature (35 and 30°C, respectively). We also quantified organic carbon sequestration in abiotic experiments with organic mixtures. We evaluated the role of mineralogy, salinity, and type of organic carbon (in biomass vs. organic mixtures without cells) in the sequestration capacity and stability of iron sulfide minerals.

## Materials and Methods

### Microorganisms and Media

*Desulfovibrio hydrothermalis* AM13 (called *Dh* AM13 hereafter) is a deep-sea marine sulfate-reducing bacterium (SRB) and grows optimally at 35°C (Alazard et al., 2003). *Dh* AM13 was purchased from DSMZ (Deutsche Sammlung von Mikroorganismen und Zellkulturen) under the reference DSM 14728. DSMZ 195c medium (2014 recipe) was used to grow *Dh* AM13. All solutions were prepared with ultrapure water. The following salts were dissolved in 870 ml water to prepare Solution A: 3.00 g Na_2_SO_4_, 0.20 g KH_2_PO_4_, 0.30 g NH_4_Cl, 21.00 g NaCl, 3.10 g MgCl_2_ × 6 H_2_O, 0.50 g KCl, 0.15 g CaCl_2_ × 2 H_2_O, and 1.0 ml selenite-tungstate solution (as described in DSMZ 385 medium). Resazurin was omitted. Solution A was gassed for at least 30 min with a N_2_/CO_2_ gas mixture and autoclaved in a 2-L bottle sealed with a thick black rubber stopper and a screw cap. A buffer solution (final volume 100 ml) was prepared with 5.00 g NaHCO_3_, gassed for at least 30 min with a N_2_/CO_2_ gas mixture, and autoclaved in a serum vial sealed with a blue butyl stopper and an aluminum cap. Solution A and the buffer were mixed in a vinyl anaerobic chamber (Coy Laboratory Products) and supplemented with 1 ml of filter-sterilized SL-10 trace element solution (as described in DSMZ 320 medium) and 10 ml of filter-sterilized vitamin solution (as described in DSMZ 141 medium). Autoclaved sodium DL-lactate syrup (60 w/w%, Sigma) was added to the medium: 5 ml (very high lactate, VHL), 3.1 ml (high lactate, HL), 1.5 ml (low lactate, LL), and 1.0 ml (very low lactate, VLL). Corresponding lactate and sulfate concentrations are given in Table 1.

**Table 1:** Cell biomass production in cultures of the two sulfate-reducing bacteria used in this study: marine strain *Desulfovibrio hydrothermalis* AM13 and freshwater strain *Desulfovibrio magneticus* RS-1.

	*Desulfovibrio hydrothermalis* AM13				*Desulfovibrio magneticus* RS-1			
	Marine medium: ~21.1 mM sulfate				Freshwater medium: ~10.0 mM sulfate			
	VLL	LL	HL	VHL	VLL	LL	HL	VHL
Lactate concentration	7.0	10.4	21.6	34.8	7.0	10.4	20.9	34.8
(mM, calculated)								
Reduced sulfate	3.5	5.2	10.8	17.4	3.5	5.2	10.0	10.0
(mM, expected)								
Reduced sulfate	2.7 ± 0.9	3.9 ± 1.2	9.8 ± 0.4	17.5 ± 1.4	4.8 ± 1.3	7.4 ± 2.1	9.8 ± 0.7	10.2 ± 0.4
(mM, measured)								
Cell biomass carbon	33.4 ± 4.0	40.7 ± 1.5	74.8 ± 3.5	106.1 ± 9.3	20.7 ± 5.2	23.1 ± 7.6	26.6 ± 0.9	31.5 ± 2.7
(mg/L, measured)								
Cell biomass carbon	2.8 ± 0.3	3.4 ± 0.1	6.2 ± 0.3	8.8 ± 0.8	1.7 ± 0.4	1.9 ± 0.6	2.2 ± 0.1	2.6 ± 0.1
(mmol/L)								
Initial molar C/Fe ratio	0.7 ± 0.2	0.9 ± 0.2	1.6 ± 0.4	2.2 ± 0.5	0.4 ± 0.1	0.5 ± 0.2	0.6 ± 0.1	0.7 ± 0.2

*VLL, LL, HL, and VHL refer to the lactate concentrations used in experiments (see “Materials and Methods”). The expected reduced sulfate concentrations were calculated using the typical stoichiometry associated with microbial sulfate reduction of 2 mol of lactate oxidized per 1 mol of sulfate reduced. Actual sulfate concentrations were determined using the barium sulfate assay as described in the Methods section. Concentrations of reduced sulfate (mM) presented here are average values from multiple experiments at a given condition. Biomass carbon concentrations in cell pellets recovered from cultures grown without Fe(II) were determined using an elemental analyzer, as described in the Methods section. Average values and standard deviations were calculated from cell pellets collected from 2 to 5 independent incubations. Biomass carbon concentrations in mg/L were converted to mol/L to calculate total molar biomass C/Fe ratios in incubations. An average concentration of 4.0 ± 0.9 mM Fe(II) was calculated from measurements done in all experiments (whether added at the beginning or at the end of experiments)*.

*Desulfovibrio magneticus* RS-1 (called *Dm* RS-1 hereafter) is a freshwater SRB and grows optimally at 30°C (Sakaguchi et al., 2002). *Dm* RS-1 came from the culture collection of Dr. Dennis Bazylinski at UNLV. A freshwater medium (Ingvorsen and Jørgensen, 1984) was used to grow *Dm* RS-1. All solutions were prepared with ultrapure water. A mineral solution (final volume 1 l after pH adjustment) contained: 1.42 g Na_2_SO_4_, 0.32 g Na_2_HPO_4_, 0.11 g KH_2_PO_4_, 0.25 g NH_4_Cl, 1.00 g NaCl, 0.6 g MgCl_2_ × 6 H_2_O, 0.20 g CaCl_2_ × 2 H_2_O, and 1.0 ml modified Wolfe’s trace element solution (as described in DSMZ 141 medium). Sodium DL-lactate syrup (60 w/w%, Sigma) was added to the mineral solution: 5 ml (very high lactate, VHL), 3.0 ml (high lactate, HL), 1.5 ml (low lactate, LL), and 1.0 ml (very low lactate, VLL). The pH of the mineral solution was adjusted to 7.1 before gassing the solution for at least 30 min with a N_2_/CO_2_ gas mixture and autoclaving it in a 2-L bottle sealed with a thick black rubber stopper and a screw cap. A buffer solution (final volume 50 ml) was prepared with 4.00 g NaHCO_3_, gassed for at least 30 min with a N_2_/CO_2_ gas mixture, and autoclaved in a serum vial sealed with a blue butyl stopper and an aluminum cap. The mineral solution and buffer were mixed in the anaerobic chamber and supplemented with 1 ml of selenite-tungstate solution (see above) and 1 ml of vitamin solution (see above). Lactate and sulfate concentrations are given in Table 1.

Both SRB couple the incomplete oxidation of lactate with sulfate reduction according to the following reaction (Rabus et al., 2013):


Eq. (1) $$\begin{array}{l} {2CH}_{3}{\mathrm{CHOHCOO}}^{-}+{{\mathrm{SO}}_{4}}^{2-}+2H^{+}\to {2CH}_{3}{COO}^{-}+\\ {2CO}_{2}+H_{2}S+2H_{2}O \end{array}$$

### Preparation of SRB Cultures for Biomass Carbon Quantification

Cultures were prepared in a vinyl anaerobic chamber (Coy Laboratory Products). Media, as described above, were dispensed in serum vials (50 ml of medium per 110-ml glass bottle) and inoculated with 250 μl from a stock culture of *Dm* RS-1 and *Dh* AM13 and incubated for ~1 month at 30°C and 35°C, respectively. Cell pellets were recovered from *Dh* AM13 cultures and from *Dm* RS-1 cultures by centrifugation at 8,000 g for 20 min. Cell pellets were then resuspended in anoxic sterile water for carbon quantification in the elemental analyzer (see below).

### Co-precipitation of Iron Sulfide Minerals With Sulfate-Reducing Bacteria and With Organic Mixtures

All experiments were prepared in the anaerobic chamber. The media, as described above, were dispensed in serum vials (50 ml of medium per 110-ml glass bottle). Fe(II) was added to each vial at a concentration of 4 mM (from a sterile anoxic 1 M FeCl_2_ solution). For each microorganism and lactate concentration, 10–20 bottles containing the Fe medium were inoculated with 250 μl from a fresh stock culture of *Dm* RS-1 and *Dh* AM13 and incubated at 30°C and 35°C, respectively (Experiments named Fe experiments). When SRB are grown in Fe medium, iron sulfide minerals tend to precipitate near cells and have large mineral particles (Picard et al., 2018). When Fe(II) is added after the bacteria have completed sulfate reduction, iron sulfide minerals precipitate away from cells, and have a small particle size (Picard et al., 2018). Therefore, for some experimental conditions (Experiments named AddFe experiments), we co-precipitated iron sulfide minerals by adding Fe(II) at a concentration of 4 mM (from a sterile anoxic 1 M FeCl_2_ solution) to fully grown cultures of *Dm* RS-1 and *Dh* AM13 after sulfate concentrations had become stable. Cultures without Fe were prepared as described in the former paragraph. After Fe(II) addition, cultures were placed back at 30°C and 35°C, respectively. At each time point, two serum vials were taken out of the incubator for analysis.

Three sets of abiotic experiments were also prepared (Table 2). Iron sulfide minerals were co-precipitated with mixtures of organic molecules (tryptone, yeast extract, and casamino acids) in the marine medium used for *Dh* AM13. Solutions of Bacto tryptone (pancreatic digest of casein), Difco yeast extract, and Bacto casamino acids (acid-hydrolyzed casein) were prepared at 50 g/l each with ultrapure water, degassed with N_2_, and autoclaved in sealed serum vials. Marine medium was dispensed in serum vials (50 ml of medium per 110-ml glass bottle). Fe(II) was added to each vial at a concentration of 4 mM (from a sterile anoxic 1 M FeCl_2_ solution). Then, 1 ml of either organic mixture was added to serum vials. The next day, 1 ml was added from a sterile anoxic sodium sulfide solution (~457 mM) to precipitate all Fe(II) as iron sulfide minerals and provide excess sulfide to the medium. A final concentration of 9.1 mM was estimated and corresponded approximately to the concentration of sulfide produced in experiments with high lactate (HL) concentrations (Picard et al., 2018). Mineral suspensions were incubated at 35°C. At each time point, two serum vials were used for analysis.

**Table 2:** Summary of experimental conditions for abiotic experiments with organic molecules.

	Abiotic with tryptone Marine medium	Abiotic with yeast extract Marine medium	Abiotic with casamino acids Marine medium
Added sulfide (mM)	9.1	9.1	9.1
Concentration of carbon from organic molecules (mg/L, measured)	428 ± 10	385 ± 4	326 ± 7
Concentration of carbon from organic molecules (mmol/L)	36.0 ± 0.8	32.1 ± 0.3	27.0 ± 0.6
Initial molar C/Fe ratio	9.0 ± 0.2	8.0 ± 0.1	6.8 ± 0.2

Iron sulfide minerals were co-precipitated in the marine medium with either tryptone, yeast extract, or casamino acids. A concentration of 4 mM Fe(II) was used in all experiments; therefore, sulfide was in excess in all conditions and all Fe(II) precipitated as iron sulfide minerals.

### Spectrophotometry

#### Quantification of Soluble Sulfate

Sulfate was quantified in bacterial cultures using the barium sulfate assay as described elsewhere (Kolmert et al., 2000). 100 μl of culture/medium collected from serum vials in the anaerobic chamber were mixed with 900 μl of zinc acetate. Samples were centrifuged at 13,000 g to remove any zinc sulfide precipitates. Supernatants were recovered and mixed with barium chloride (~60 mg) using a vortex mixer. 1 ml of conditioning reagent was added to each sample. The conditioning reagent was prepared with 150 g NaCl, 100 ml pure glycerol, 60 ml of concentrated HCl, and 200 ml of 95% ethanol. Ultrapure water was added to produce 1 l of reagent. Turbidity produced by barium sulfate precipitates was monitored at 420 nm on a Beckman Coulter™ DU-530 spectrophotometer. Sulfate concentrations were determined using a calibration curve prepared with sodium sulfate solutions.

#### Quantification of Total Ferrous Iron

Total Fe(II) was quantified in experiments using the ferrozine assay (Stookey, 1970). 100 μl of culture collected from serum vials in the anaerobic chamber were mixed with 900 μl of HCl 1 M, which completely dissolved biogenic and abiotic iron sulfide minerals produced in all experiments. 25 μl of the fixed sample was mixed with 975 μl of ferrozine solution, prepared at a final concentration of 0.1% ferrozine in HEPES buffer at pH 7.0. Absorbance was measured at 563 nm after 5 min. Fe(II) concentrations were determined using a calibration curve prepared with ammonium ferrous sulfate solutions.

### Solid Phase Preparation for CN Quantification and for *X*-Ray Diffraction

Solid phases were recovered from biomineralization experiments and from abiotic experiments on a regular basis over the course of long-term incubations. For each time point, solid phases were prepared from two different serum vials. Cultures containing minerals and abiotic mineral suspensions (each 50 ml) were transferred to Nalgene tubes sealed with O-ring screw caps in the anaerobic chamber and centrifuged at 8,000 g for 30 min at room temperature. Solid phases were washed with sterile anoxic water in the anaerobic chamber and left to dry afterwards.

### Elemental Analysis (Carbon and Nitrogen)

Total carbon (TC), total nitrogen (TN), and CN ratios were determined in solid phases recovered from biomineralization and abiotic mineralization experiments, in cell pellets recovered from cultures grown without Fe, and in stock solutions of organic molecules (used for abiotic experiments) using an Elementar varioMAX analyzer in the CN mode. Dried solid phases were gently ground in an agate mortar and pestle in the anaerobic chamber and transported to the instrument in air-tight jars. Samples were weighted in stainless steel crucibles just before analysis and loaded into the instrument. Limited air contact with dried minerals did not impact their TC and TN contents. Cell pellets were resuspended in anoxic ultrapure water and transferred to stainless steel crucibles just before analysis. The instrument was calibrated with >99.0% pure L-glutamic acid (Merck). The stability of the instrument was monitored by measuring small amounts of standard materials with a range of C and N contents: low organic content soil standard OAS (Elemental microanalysis, C: 1.86%, N: 0.122%), reagent grade > 98% L-aspartic acid (Sigma, C: 36.10%, N: 10.52%), and Buffalo River sediment standard (NIST 8704, C: 3.351%, N: 0.19%).

### *X*-Ray Diffraction

Powder *X*-ray diffraction was used to determine the mineralogy of solid phases, prepared as described above. Dried minerals were homogenized in 200-proof ethanol using an agate mortar and pestle and subsequently mounted on a zero-diffraction silicon plate (MTI corporation) in the anaerobic chamber. Samples were transported to the instrument in air-tight jars. Powder *X*-ray diffraction (XRD) data were acquired with a Proto AXRD at the Geoscience department of the University of Nevada Las Vegas. *X*-ray diffractograms were acquired using Cu K-α radiation (30 kV-20 mA), between 10° and 60° 2θ angles with increment steps of 0.05° and a dwell time of 1 s.

### Experimental Design, Data Processing, and Statistical Tests

Concentrations of cell biomass carbon (Table 1) are average values from 2 to 5 cultures grown without Fe(II) for ~1 month. Errors represent ± one standard deviation. For each condition tested, biomineralization experiments consisted of up to 20 replicate serum vials prepared with the same batch of medium, the same solution of FeCl_2_ and inoculated with the same stock culture (or prepared with the same sulfide solution for abiotic experiments). At each time point, two serum vials were sacrificed to collect liquid samples and minerals; and measured TC, TN and CN represent average values from these two independent samples. Errors represent ± one standard deviation. For each given experimental condition, the concentration of reduced sulfate presented in Table 1 is an average value of all time points taken after 2 weeks of incubation. The average concentration of 4.0 ± 0.9 mM Fe(II) was calculated from all time points taken in this study. Stabilized data of TC, TN, and CN ratio presented in Table 3 were calculated by averaging values measured over a period of time during which the values appeared stable. To determine the time at which the data became stable for a given experimental condition, i.e., the time at which values do not change significantly anymore, a one-way ANOVA analysis with a Tukey HSD test was performed to compare data collected at each time point against one other. We used the same range of time to calculate stabilized data for TN contents and for CN rations. To evaluate differences between TC% in iron sulfide minerals co-precipitated with bacteria in cultures grown with Fe(II), e.g. Fe experiments in Table 3, and in iron sulfide minerals co-precipitated with bacteria in cultures where Fe(II) was added after growth happened (AddFe experiments), when available, a Student unpaired *t*-test was performed between stabilized values (See Table 3 to see under which conditions AddFe experiments have been performed). Total carbon concentrations removed from medium (Table 3) were calculated by assuming that all 4 mM of Fe(II) precipitated as FeS (mackinawite), therefore producing an average concentration of mackinawite of 352 mg/l. Although in some conditions, Fe(II) was slightly in excess and in some other conditions, greigite was also produced, total carbon concentrations removed from the medium would fall in the same range.

**Table 3:** Summary of parameters characterized in iron sulfide minerals: total carbon (TC) content (% w/w), total nitrogen (TN) content (% w/w), C/N ratio, TC content (mg/g mineral), TC sequestered from medium (mg/L and %), and mineralogy.

	Stabilized TC (w/w %)	Stabilized TN (w/w %)	Stabilized C/N ratio	Stabilized TC (mg/g = g/kg)	TC removed from medium (mg/L)	TC removed from medium (%)	Mineralogy
Biotic experiments – *Desulfovibrio hydrothermalis* AM13 (marine medium)							
*Dh* **Fe** VLL (all)	3.7 ± 0.3	1.5 ± 0.4	2.7 ± 0.7	37 ± 3	14 ± 1	42	Mackinawite^†^
*Dh* **AddFe** VLL (2 mo)	4.1 ± 0.3	1.2 ± 0.1	3.5 ± 0.1	41 ± 3	15 ± 1	45	ND
*Dh* **Fe** LL (all)	5.0 ± 0.6	1.2 ± 0.2	4.2 ± 0.7	50 ± 6	18 ± 2	44	Mackinawite^†^
*Dh* **AddFe** LL (all)	5.6 ± 1.8	1.8 ± 0.6	3.1 ± 0.7	56 ± 18	21 ± 7	51	ND
*Dh* **Fe** HL (>2 mo)	8.3 ± 0.4	2.3 ± 0.6	3.8 ± 0.8	83 ± 4	32 ± 2	43	Mackinawite^*†^ Greigite^*†^
*Dh* **AddFe** HL	ND	ND	ND	ND	ND	ND	Mackinawite^*^
*Dh* **Fe** VHL (>2 mo)	13.5 ± 0.7	3.3 ± 0.7	4.2 ± 0.7	135 ± 7	55 ± 3	52	Mackinawite^†^ Greigite^†^
*Dh* **AddFe** VHL (>2 mo)	11.0 ± 0.6	2.7 ± 0.2	4.1 ± 0.1	110 ± 6	44 ± 2	42	ND
Biotic experiments – *Desulfovibrio magneticus* RS-1 (freshwater medium)							
*Dm* **Fe** VLL (>1 mo)	3.7 ± 0.3	0.9 ± 0.1	4.1 ± 0.3	37 ± 3	14 ± 1	67	Mackinawite^†^ Greigite^†^
*Dm* **AddFe** VLL	ND	ND	ND	ND	ND	ND	ND
*Dm* **Fe** LL (all)	6.1 ± 1.0	1.8 ± 0.6	3.7 ± 0.7	61 ± 10	23 ± 4	100	Mackinawite^†^ Greigite^†^
*Dm* **AddFe** LL	ND	ND	ND	ND	ND	ND	ND
*Dm* **Fe** HL (all)	9.5 ± 0.7	2.5 ± 0.4	3.8 ± 0.6	95 ± 7	37 ± 3	137	Mackinawite^†^
*Dm* **AddFe** HL (>2 mo)	7.5 ± 2.3	1.5 ± 0.7	5.2 ± 1.1	75 ± 23	29 ± 9	107	ND
*Dm* **Fe** VHL (all)	10.2 ± 1.2	2.3 ± 0.9	3.6 ± 0.9	102 ± 12	40 ± 4	125	Mackinawite^†^
*Dm* **AddFe** VHL	ND	ND	ND	ND	ND	ND	ND
Abiotic experiments – organic mixtures (marine medium)							
Abio Tryptone (6 mo)	5.1 ± 0.6	2.3 ± 0.7	2.5 ± 0.8	51 ± 6	19 ± 3	4	Mackinawite^#^
Abio Yeast extract (6 mo)	5.2 ± 0.8	2.3 ± 1.0	2.5 ± 0.6	52 ± 8	19 ± 2	5	Mackinawite^#^
Abio Amino acids (6 mo)	1.2 ± 0.1	1.1 ± 0.0	1.0 ± 0.1	12 ± 1	4 ± 1	1	Mackinawite^#^

Iron sulfide minerals were precipitated in the presence of sulfate-reducing bacteria Desulfovibrio hydrothermalis AM13 (Dh) and Desulfovibrio magneticus RS-1 (Dm; Biotic experiments); in the presence of organic mixtures, e.g., tryptone, yeast extract, and amino acids (Abiotic experiments). “Fe” experiments refer to experiments in which Fe(II) was added to the medium before inoculation of SRB, while “AddFe” experiments refer to experiments in which Fe(II) was added to fully grown SRB cultures. “Stabilized” data correspond to average values of data after values have reached a stable level (data points included in the calculation are indicated between brackets near the experiment name). Fe(II) was added at a concentration of 4 mM in all experiments, therefore producing an average concentration of iron sulfide minerals of 352 mg/l in all experiments. Mineralogy results from Picard et al., 2018^*^, from Picard et al., 2021^#^, and from this study^†^.

## Results and discussion

Organo-mineral interactions play an important role in the long-term preservation of organic carbon in a variety of environments, e.g., permafrost, soils, and sediments (Solomon et al., 2012; Keil and Mayer, 2014; Kleber et al., 2015, 2021; Opfergelt, 2020; Wagai et al., 2020). While the majority of minerals in sediments and soils is represented by clays and Fe/Mn oxides (Kleber et al., 2021), there is a lack of understanding of the role of authigenic minerals forming in anoxic environments in the sequestration of organic carbon (OC). In this study, we quantified the OC sequestration capacity of iron sulfide minerals in long-term laboratory experiments. As sulfide production in low-temperature environments is driven by microbial sulfate reduction, we co-precipitated biogenic iron sulfide minerals in marine and freshwater media with varying amounts of biomass from sulfate-reducing bacteria (SRB) and monitored their carbon and nitrogen contents in long-term (>1 year) incubations. We discuss the role of salinity, cells, and mineralogy in building strong and stable organo-mineral associations.

### Biomass Carbon Production Varies Between Strains of Sulfate-Reducing Bacteria

**Figure 1:**
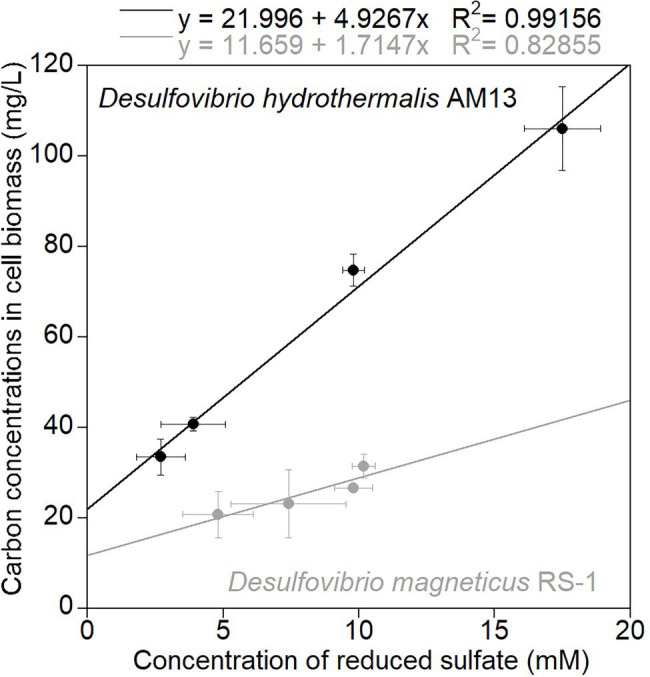
Average carbon concentrations in cell biomass (mg/L) measured in cell pellets recovered from cultures grown without Fe(II), as a function of average concentrations of reduced sulfate (mM). Black circles are data for marine strain *Desulfovibrio hydrothermalis* AM13, and gray circles are data for freshwater *Desulfovibrio magneticus* RS-1. Error bars represent ±one standard deviation. The slope of the linear regressions gives the amount of biomass carbon (g) produced per mol sulfate reduced as: 4.9 g biomass carbon/mol sulfate for *Dh* AM13 and 1.7 g biomass carbon/mol sulfate for *Dm* RS-1.

Cultures of sulfate-reducing bacteria (SRB) were grown with a fixed sulfate concentration (21.1 mM in the marine medium and 10.0 mM in the freshwater medium) and four different lactate concentrations (7.0 mM VLL, 10.4 mM LL, 20.9–21.6 mM HL, and 34.8 mM VHL) to vary amounts of sulfate reduced and therefore to vary biomass concentrations produced. In cultures of the marine strain *Desulfovibrio hydrothermalis* AM13, lactate was limiting in all conditions, and sulfate was leftover in all conditions (Table 1). In cultures of the freshwater strain *Desulfovibrio magneticus* RS-1, lactate was limiting in VLL and LL conditions, while sulfate was limiting and lactate was leftover in HL and VHL conditions (Table 1). A concentration of 4 mM Fe(II) was used in all biomineralization experiments; therefore, Fe(II) was the limiting factor to produce iron sulfide minerals. Sulfide was in excess in HL and VHL incubations with *Dh* AM13 and in all incubations with *Dm* RS-1. Although the experimental conditions were intended to produce sulfide in excess in all experiments with *Dh* AM13, sulfide was slightly limiting in VLL and LL conditions (Table 1). Cell biomass carbon concentrations, determined in cell pellets, increased with increasing sulfate reduced (Figure 1; Table 1). For *Dm* RS-1 in HL and VHL conditions, biomass concentrations were similar. An increase in the lactate concentration (in VHL vs. HL) did not impact biomass concentrations as the same amount of sulfate was reduced in these two conditions. The biomass yield differed between the two bacteria, with 4.9 g biomass carbon per mol sulfate for *Dh* AM13 and 1.7 g biomass carbon per mol sulfate for *Dm* RS-1. This converts to 9.8 g dry biomass per mol sulfate and 3.4 g dry biomass per mol sulfate, respectively, as the content of carbon of microbial biomass is ≈50% (Bar-On et al., 2018). Biomass carbon yields (typically reported as dry biomass yields in the literature) of other *Desulfovibrio* strains vary between 3.6 g and 13.5 g dry biomass per mol sulfate (with lactate as source of carbon and energy; Table 4; Traore et al., 1981, 1982; Ingvorsen and Jørgensen, 1984; Okabe and Characklis, 1992; Cooney et al., 1996). Differences in biomass carbon yields observed between different strains of *Desulfovibrio* do not appear to be related to the origin of the strain, as they range from 3.4 to 13.5 g dry biomass per mol sulfate for freshwater strains and from 3.6 to 9.8 g dry biomass per mol sulfate for marine strains. Instead, if all lactate is used for microbial sulfate reduction, differences in biomass yields between strains can occur as a result of variations between ATP generation, and/or variations in ATP utilization by anaerobic synthesis (Traore et al., 1982).

**Table 4:** Estimated biomass carbon and dry biomass yields of *Desulfovibrio hydrothermalis* AM13 and *Desulfovibrio magneticus* RS-1 grown with sulfate and lactate.

	Biomass carbon (g/mol sulfate)	Dry biomass (g/mol sulfate)	References
*D. hydrothermalis* AM13 (m)	4.9	9.8	This study
*D. africanus* (m)		3.6	Traore et al., 1982
*D. sapovorans* (m)		8.7	Ingvorsen and Jørgensen, 1984
*D. salexigens* (m)		8.0	Ingvorsen and Jørgensen, 1984
*D. magneticus* RS-1 (fr)	1.7	3.4	This study
*D. desulfuricans* Essex 6 (fr)		10.8	Cooney et al., 1996
*D. desulfuricans* Norway 4 (fr)		8.2	Traore et al., 1982
*D. gigas* (fr)		7.4	Traore et al., 1982
*D. vulgaris* (fr)		13.5	Traore et al., 1981
*D. vulgaris Marburg* (fr)		7.1	Ingvorsen and Jørgensen, 1984
*D. desulfuricans* (fr)		5.4	Okabe and Characklis, 1992

Values are compared with those reported for other *Desulfovibrio* species. (fr) and (*m*) indicate freshwater and marine strains, respectively.

### The Amount of Carbon and Nitrogen Sequestered by Biogenic Iron Sulfide Minerals Increases With Increasing Biomass Concentrations in the Media

**Figure 2:**
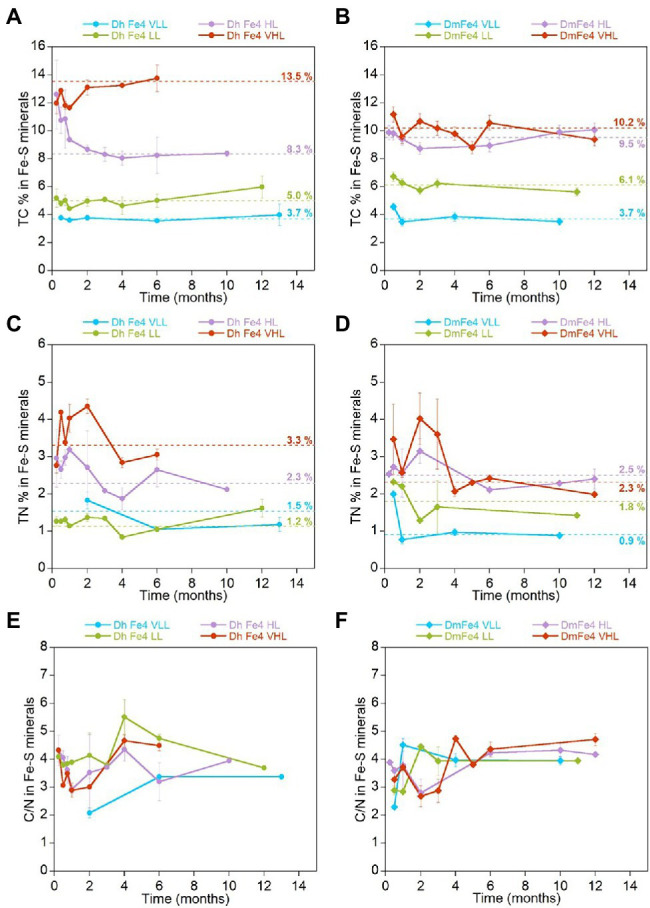
Evolution of the average total carbon (TC) content (w/w %), average total nitrogen (TN) content (w/w %), and average C/N ratio in iron sulfide minerals precipitated in the presence of *Desulfovibrio hydrothermalis* AM13 **(A,C,E)** and in the presence of *Desulfovibrio magneticus* RS-1 **(B,D,F)** as a function of time. Error bars represent ±one standard deviation. Incubations were performed with four different initial lactate concentrations (VLL, LL, HL, and VHL) which controlled the amount of sulfate reduced and biomass produced (Table 1; Figure 1). Results presented here are from minerals produced in cultures that were grown in the presence of Fe(II). Average values of stabilized levels of TC and TN (see Table 3 for data points encompassed in stabilized values) are indicated over dashed lines **(A-D)**. TC and TN contents in iron sulfide minerals increase with increasing lactate concentrations in the medium (corresponding to increasing concentrations of reduced sulfate, i.e., increasing concentrations of cell biomass). CN ratios stabilize to a narrow range of values for both bacteria: 2.7–4.2 for marine *Dh* AM13and 3.6–4.1 for freshwater *Dm* RS-1 **(E, F)**.

Biogenic minerals precipitated in cultures of freshwater and marine SRB grown with Fe(II) incorporated carbon and nitrogen from the media that contained microbial biomass (Figure 2). When Fe(II) is added to the HL medium before inoculation of *Dh* AM13, iron sulfide minerals precipitate at the surface of cells and form crusts closely associated with cells, but also have a larger particle size than abiotic minerals (Picard et al., 2018). Total carbon (TC) contents of iron sulfide minerals increased with increasing initial lactate concentrations (i.e., with increasing concentrations of reduced sulfate *and* increasing concentrations of produced biomass; *Dh*, Figure 2A; *Dm* Figure 2B, Fe experiments in Table 3). In very low lactate (VLL) conditions, TC levels were stable early on and reached their stabilized levels of 3.7 ± 0.3 w/w% for *Dh* AM13 and for *Dm* RS-1 after 2 weeks and 1 month of incubation, respectively. The same was observed for low lactate (LL) conditions, in which TC levels reached their stable levels of 5.0 ± 0.6 w/w% for *Dh* AM13 and of 6.1 ± 1.0 w/w% for *Dm* RS-1 after 2 weeks and 1 week of incubation, respectively. In cultures of *Dh* AM13 grown in HL medium, the TC content of minerals decreased sharply from ~12.6 ± 2.4 w/w % after 1 week of incubation to reach a stable level of 8.3 ± 0.4 w/w% after 2 months of incubation. TC levels in biogenic minerals precipitated with *Dh* AM13 in VHL medium fluctuated within the first 2 months of incubation before reaching a stable level of 13.5 ± 0.7 w/w%. Minerals older than 6 months produced in VHL medium with *Dh* AM13 were lost due to a malfunction of the elemental analyzer. Based on the observation that TC levels were already stable after 6 months in all other conditions (with both SRB), we assumed that this was also the case for minerals precipitated with *Dh* AM13 in VHL medium (Figure 2A). In minerals precipitated in HL and VHL conditions with *Dm* RS-1, TC levels were similar and reached stabilized values of 9.5 ± 0.7 w/w% after 1 week of incubation and 10.2 ± 1.2 w/w% after 2 weeks of incubation, respectively. In both conditions, similar amounts of sulfate were reduced and similar amounts of biomass were produced (Figures 1, 2B; Table 1). Total nitrogen (TN) contents in iron sulfide minerals showed more variability over time, but overall followed similar trends to TC contents (Figures 2C,D). Stabilized C/N ratios of minerals converged to a narrow range of 2.7–4.2 for minerals produced with marine *Dh* AM13 (Figure 2E), and of 3.6–4.1 for minerals produced with freshwater *Dm* RS-1 (Figure 2F).

Additionally, we measured TC and TN levels, and CN ratios of biogenic minerals precipitated by adding Fe(II) to fully grown cultures of *Dh* AM13 and *Dm* RS-1 in selected conditions (AddFe experiments in Table 3). When adding Fe(II) to cultures of *Dh* AM13 after full growth in HL medium, minerals tend to precipitate away from cells and have a small particle size similar to this produced without bacteria (Picard et al., 2018). For minerals produced in VLL and LL conditions with *Dh* AM13, average values of TC were not significantly different in Fe and AddFe experiments (*p* > 0.05). In minerals produced in HL conditions with *Dm* RS-1 and in VHL conditions with *Dh* AM13, average values of TC were significantly different in Fe and AddFe experiments (*p* < 0.05; Table 3). When cells are grown with Fe(II), the precipitation process occurs over several days. There is the possibility for Fe(II) to interact with the surface of microbial cells, as there is a strong affinity between cations, such as Fe(II), and negatively charged bacterial cell surfaces (Ferris et al., 1987; Beveridge, 1989). As cells grow and produce sulfide, mackinawite precipitates at the surface of cells, while new cells can interact with leftover Fe(II). In contrast, when Fe(II) is added all at once after growth and sulfide production, precipitation of mackinawite is very fast. In conditions producing the highest biomass contents (HL and VHL conditions), the slower process of precipitation might allow for sequestering more biomass.

We previously used scanning transmission *X*-ray microscopy (STXM) coupled with near-edge *X*-ray absorption fine structure spectroscopy (NEXAFS) to determine the type of carbon compounds that can associate with biogenic iron sulfide minerals produced in the presence of *Dh* AM13 in HL medium, whether Fe(II) was provided before inoculation or after growth of the SRB (Picard et al., 2019, 2021). The NEXAFS spectra at the C K-edge of biogenic iron sulfide minerals were deconvolved with standard spectra of proteins, polysaccharides, and lipids and were similar to the C K-edge spectra of non-encrusted bacterial cells reported in other studies (Cosmidis et al., 2015; Picard et al., 2019). The NEXAFS spectra at the N K-edge of biogenic iron sulfide minerals were indicative of fresh proteinaceous material (Picard et al., 2019). Inorganic carbon (e.g., from the carbonate buffer of the medium) and inorganic nitrogen (added as NH_4_Cl to the medium) did not contribute significantly to the spectroscopic signatures of iron sulfide minerals (Picard et al., 2019, 2021). Therefore, TC and TN levels in the present study reflect total organic carbon (TOC) and total organic nitrogen (TON) levels in biogenic iron sulfide minerals. Additionally, the spectroscopic signature of organic carbon associated with biogenic iron sulfide minerals did not change over the course of long-term experiments, suggesting no overall partitioning of organic molecules (Picard et al., 2019, 2021). In the present study, we are confirming the quantitative preservation of microbial biomass in iron sulfide mineral aggregates over long periods of time.

While the interactions between iron sulfide minerals (e.g., mackinawite) and heavy metals and pollutants have been extensively studied in the context of bioremediation applications (Chen et al., 2019), to our best knowledge, only two studies have explored TC levels in iron sulfide minerals in laboratory incubations (Balind, 2019; Wang et al., 2019). In experiments co-precipitating mackinawite with dissolved organic carbon extracted with water from soil, corn, and phytoplankton, levels of TOC in mackinawite of up to ~80 mg/g, ~105 mg/g, and ~ 145 mg/g were measured, respectively. In sorption experiments with the above compounds, levels of TOC in mackinawite were slightly lower and reached levels up to ~45 mg/g, ~65 mg/g, and ~ 90 mg/g, respectively (Balind, 2019). Overall, this is comparable to our experiments co-precipitating iron sulfide minerals with biomass (up to ~135 mg/g with *Dh* AM13 and up to ~102 mg/g with *Dm* RS-1). In sorption experiments of commercially available iron sulfide FeS and pyrite FeS_2_ with dissolved organic matter extracted with water from a peat, TOC levels reached levels up to 1.4 mg/g in FeS and up to 0.065 mg/g in pyrite (Wang et al., 2019). The differences in TOC levels in the commercially available FeS and freshly precipitated nanocrystalline mackinawite might come from the different nature and crystallinity of minerals. The commercially available FeS might not be mackinawite, therefore preventing comparison with our results and those of Balind (2019). Iron sulfide minerals are less abundant than other types of minerals known to preserve organic carbon [e.g., Fe(III) oxides or clay minerals]; however, they are likely to closely interact with cell biomass when precipitated in anoxic environments. Indeed, in most anoxic environments at the surface of the Earth, microbial sulfate reduction is the source of 97% of sulfide (Rickard, 2012a; Picard et al., 2016); therefore, precipitation of iron sulfide minerals is likely to happen in close proximity to SRB cells. The following is an attempt at estimating the global amount of organic carbon that could potentially be sequestered by biogenic iron sulfide minerals in marine sediments. Estimates of global rates of microbial sulfate reduction in marine sediments vary (Jørgensen, 2021). For the purpose of a rough estimate, we use the lowest overall estimate of 11.3 Tmol of sulfate reduced yearly in marine sediments (Bowles et al., 2014). Not all sulfide produced ends up as iron sulfide minerals; sulfide can interact with organic matter, can be reoxidized to sulfate or intermediate sulfur species, and can precipitate as metastable iron sulfide minerals that will eventually transform to pyrite (FeS_2_; Jørgensen, 2021). Of the 11.3 Tmol sulfide produced per year, about 90% are reoxidized (Jørgensen, 2021), therefore leaving ~1 Tmol of sulfide per year that precipitate as iron sulfide minerals. As the first product of interaction between sulfide and ferrous iron is FeS (either as clusters, colloids, or metastable mackinawite; Rickard and Luther, 2007), this gives us an estimated production of 88 Tg of FeS per year. Based on TC measurements done in minerals precipitated in the marine medium with *Dh* AM13 (37–135 mg/g), this translates to 3–12 Tg of organic carbon that can be potentially sequestered by biogenic metastable iron sulfide minerals yearly. Considering our biomass yield of 4.9 g per mol of sulfate for *Dh* AM13, global microbial sulfate reduction would produce ~55 Tg of biomass per year. Obviously, our measurements do not reflect the reality of microbial sulfate reduction in natural environments, as cultures are grown with lactate; however, this illustrates the possibility that enough biomass would be produced to be sequestered by iron sulfide minerals in the range of 3–12 Tg. Interestingly, the global pyrite (FeS_2_) formation rate was estimated at ~5 Tg per year in marine sediments (Rickard and Luther, 2007). If metastable iron sulfide minerals are precursors to pyrite formation, the process involves dissolution before reprecipitation as pyrite (Rickard, 2012b), and this would potentially involve some loss of OC. While mineral-associated TC levels obtained in laboratory experiments are not necessarily representative of natural environments, there is recent direct evidence that iron sulfide minerals could be associated with significant amounts of OC in the environment and that it is useful to understand the basic mechanisms of interactions between OC and iron sulfide minerals (Barber et al., 2017; Balind, 2019).

### Biogenic Iron Sulfide Minerals Sequester Organic C and N More Efficiently in Freshwater Medium

**Figure 3:**
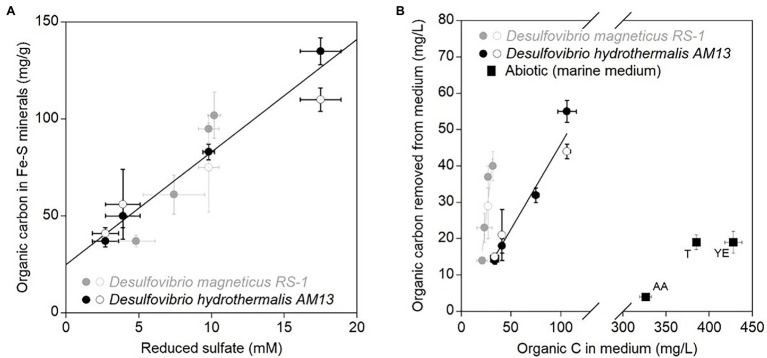
**(A)** Average stabilized organic carbon contents (mg/g) in biogenic iron sulfide minerals as a function of average concentrations of reduced sulfate (mM). Black circles are data for marine strain *Desulfovibrio hydrothermalis* AM13, and gray circles are data for freshwater *Desulfovibrio magneticus* RS-1. Closed circles are from minerals precipitated in cultures grown with Fe(II) and open circles are from minerals precipitated in cultures to which Fe(II) was added after growth of SRB. Error bars represent ±one standard deviation. The linear regression is calculated for all data points (*R*^2^ = 0.89). **(B)** Average organic carbon concentrations (mg/L) removed from the medium in biotic and abiotic experiments by iron sulfide minerals as a function of average organic concentrations of carbon (mg/L) available in the medium. Black circles are data for marine *Dh* AM13, and gray circles are data for freshwater *Dm* RS-1. Closed circles are from minerals precipitated in cultures grown with Fe(II) and open circles are from minerals precipitated in cultures to which Fe(II) was added after growth of SRB. The black linear regression is calculated for all data points from experiments with marine *Dh* AM13 (*R*^2^ = 0.95) and the gray linear regression is calculated for all data points from experiments with marine *Dm* RS-1 (*R*^2^ = 0.89). Black squares are data from abiotic experiments to which organic mixtures have been used for co-precipitation of minerals: T, tryptone, YE, Yeast extract, and AA, Casamino acids. Error bars represent ±one standard deviation. Iron sulfide minerals have a higher affinity toward organic molecules in freshwater medium than in marine medium. Iron sulfide minerals also have a higher affinity toward cell biomass than organic mixtures in abiotic conditions.

Stabilized TC levels in iron sulfide minerals produced in freshwater and marine media were correlated with concentrations of reduced sulfate (Figure 3A). However, for similar concentrations of reduced sulfate, different cell biomass concentrations were produced by each bacterium (Figure 1; Table 1). Therefore, we compared the OC removal capacity of iron sulfide minerals in freshwater and marine conditions. In all experiments, a fixed Fe(II) concentration of 4 mM was used. Assuming that all Fe(II) precipitated as iron sulfide minerals, then the same amount of iron sulfide minerals (~352 mg/l) was produced in all experiments. Using values of TC% in iron sulfide minerals, we estimated organic carbon concentrations removed from the medium (Figure 3B; Table 3). Biogenic iron sulfide minerals produced in marine conditions with *Dh* AM13 captured 42, 45, 44, and 51% of the cell biomass carbon available in VLL, LL, HL, and VHL conditions, respectively. Biogenic iron sulfide minerals precipitated in freshwater conditions with *Dm* RS-1 sequestered 67, 100, 137, and 107% of the available carbon in VLL, LL, HL, and VHL conditions, respectively. *Dm* RS-1 experiments in HL and VHL conditions showed a removal higher than 100%. We hypothesize that this might be due to uncertainties in measuring cell biomass carbon contents in pellets. While SRB can produce extracellular polymeric substances (EPS), their quantification was not possible and we assumed that they would not contribute significantly to organic carbon concentrations. In our incubations, we did not reach saturation levels; therefore, iron sulfide minerals could possibly bind more biomass than the levels we tested in this study. Due to uncertainties measuring biomass concentrations and therefore calculating organic concentrations at equilibrium in the freshwater experiments, we did not attempt to fit our data to adsorption isotherm models. Overall, our data indicate that sequestration of organic matter by iron sulfide minerals is more efficient in freshwater than in marine conditions. In a recent study investigating interactions between natural OM into ferrihydrite, a Fe(III) oxyhydroxide, it was shown that OM adsorbed more on ferrihydrite in low- and mid-ionic strength water (e.g., proxy for freshwater) than in high-ionic strength water (e.g., proxy for seawater). At high-ionic strength, Na^+^ ions seem to occupy the surface of minerals and prevent adsorption of organic matter (Tomaszewski et al., 2021).

### Cells Play an Important Role in Increasing the TC Contents of Organo-Mineral Aggregates

Abiotic iron sulfide minerals were precipitated in the marine medium with organic mixtures. The amino acid mixture (Casamino acids) and tryptone are mostly made of proteinaceous material, while yeast extract is close to the overall composition of bacteria, with the difference that yeast cells have lysed. TC levels reached 1.2 ± 0.1 w/w%, 5.1 ± 0.6 w/w%, and 5.2 ± 0.8 w/w% after 6 months of incubation with casamino acids, tryptone, and yeast extract, respectively. Despite initial concentrations of organic C in the medium three to ten times higher in abiotic experiments than in biotic experiments, and initial molar C/Fe ratios much higher in abiotic experiments, the removal of organic molecules in the medium by iron sulfide minerals was low in abiotic experiments: 6% in incubations with tryptone, 4% in incubations with yeast extract, and 1% in incubations with casamino acids (Tables 2 and 3; Figure 3B). Our previous spectroscopic study indicated that iron sulfide minerals have affinity for large proteinaceous material (from tryptone and yeast extract) and low affinity for sugars (mannose and glucose; Picard et al., 2021). The results of the present study indicate that presence of cells, and/or cellular structure and architecture play a role in increasing the amount of organic carbon sequestered through organo-mineral associations. This might be explained by the fact that when iron sulfide minerals bind to organic molecules at the surface of cells, the whole cells are preserved in mineral aggregates rather than just the molecules binding to minerals. This is supported by STXM analyses of iron sulfide minerals produced in laboratory experiments. While organic carbon is homogeneously distributed on/in abiotic minerals precipitated with tryptone and yeast extract, in biogenic minerals precipitated with *Dh* AM13 grown with Fe(II) or in cultures to which Fe(II) was added after full growth of SRB, organic carbon has a heterogeneous distribution (Picard et al., 2019, 2021). Indeed, hot spots of organic carbon (i.e., cells) are preserved in iron sulfide mineral aggregates, whether cells are encrusted in minerals or not, and these hot spots are still present after 2 years of incubation (Picard et al., 2019, 2021). The reported patchy occurrence of organic matter in continental margin sediments could therefore be explained by the abundant presence of microbial cells in sedimentary environments (Ransom et al., 1997, 1999). Interestingly, when mackinawite was precipitated with natural organic matter (NOM) extracted with water from phytoplankton, soil, or corn, the TC% in minerals reached levels as high or higher than those in minerals precipitated with SRB in our study (Balind, 2019). The NOM solutions used by Balind were filtered with 0.7 μm filters, which might leave residues of cells or smaller cells go through in the solutions, explaining how higher retention of OC might be possible. The study by Balind also highlights that iron sulfide minerals have a high affinity toward natural OM (Balind, 2019).

### Mineralogical Changes Do Not Play an Apparent Role in the Stability of TC and TN Contents in Iron Sulfide Minerals

**Figure 4:**
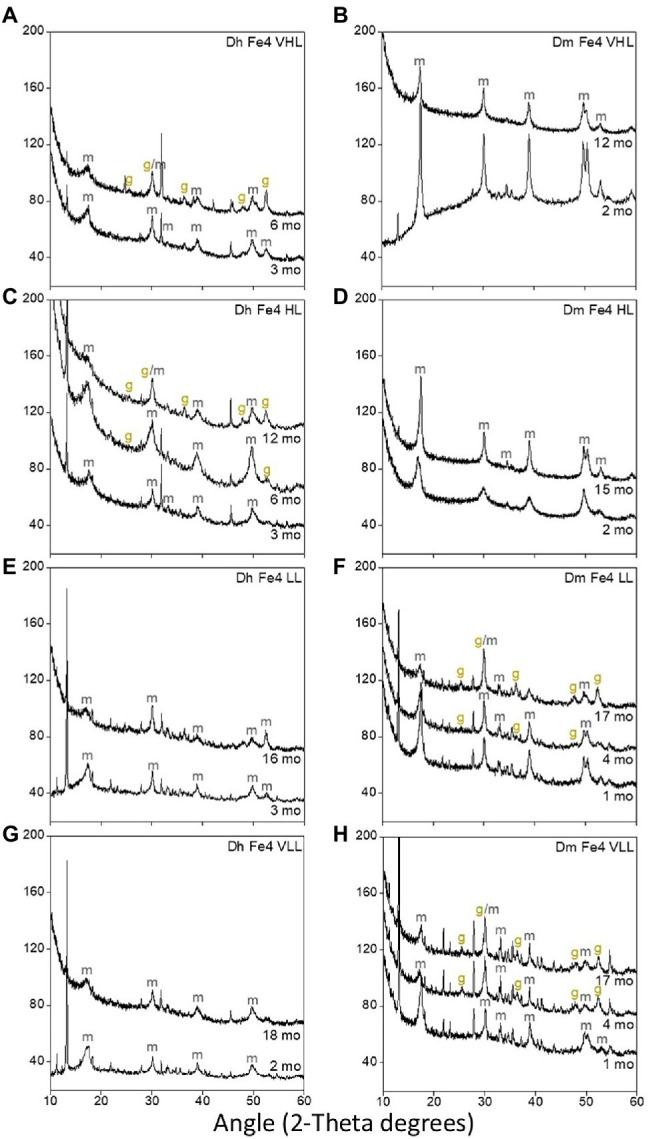
*X*-ray diffraction (XRD) spectra of biogenic minerals precipitated in cultures of Dh AM13 **(A,C,E,G)** and in cultures of Dm RS-1 **(B,D,F,H)**. Only the peaks relevant to iron sulfide minerals have been labeled (*m*, mackinawite; g, greigite). Other peaks visible on theses spectra are form vivianite [Fe(II) phosphate mineral].

*Dh* AM13 can influence iron sulfide mineral transformations. Notably, when *Dh* AM13 is grown in HL medium in the presence of Fe(II), mackinawite starts transforming to greigite *via* solid-state transformation after 5 months of incubation, while no transformation is observed in abiotic experiments and in biotic experiments to which Fe(II) is added after full growth of the bacteria (Picard et al., 2018). Mackinawite to greigite transformation was also detected in cultures of *Desulfovibrio vulgaris* grown with Fe(II) grown with Fe(II) after several months of incubation (Mansor et al., 2019). As TC contents in minerals precipitated with marine *Dh* AM13 in HL conditions stabilized after 2 months of incubation, just before mineral transformations started to be measurable by *X*-ray diffractions (XRD), we hypothesized that mineral transformations could potentially influence TC contents in minerals and their long-term stability. We therefore characterized the mineralogy of iron sulfide minerals in all conditions at relevant times during long-term incubations (Figure 4). Transformation of mackinawite to greigite was observed, but not in all conditions. In *Dh* cultures, greigite was produced in VHL and HL conditions, while it was not detected when experiments started with LL and VLL concentrations. While we previously started to observe intense peaks representative of greigite in the XRD spectra collected from 5-month-old minerals (Figure 1 in Picard et al., 2018), we only saw traces of greigite after 6 months of incubation in the present study (Figure 4). The same medium was used in both studies, the main difference was that only 1 ml of trace element solution was used in the preparation of media for this study, while 10 ml was used in the former study. This potentially indicates that the microbial influence on mineral formation and transformation could be influenced, directly or indirectly, by the availability of trace elements. However, this observation will require further exploration. In *Dm* cultures, we also observed different outcomes in mineralogical products depending on the starting concentration of lactate. Greigite was detected in LL and VLL conditions, while it was not detected in HL and VHL conditions. We did not detect pyrite in any of the conditions. The absence of pyrite might be explained by the fact that the presence of organic carbon in iron sulfide precipitation experiments seems to inhibit pyrite formation and promote greigite formation (Morse and Wang, 1997; Rickard et al., 2001). However, we were not able to identify parameters that control greigite formation and/or prevent mackinawite transformation in our study. Additional work would be needed for that purpose. In conclusion, mineralogical changes cannot be attributed to changes in TC levels.

## Conclusion

Climate change will reduce oxygen contents in the ocean and will expand oxygen minimum zones, which will modify organic carbon delivery to sediments (Keil, 2017). Authigenic iron sulfide minerals have the potential to play a role in preserving significant amounts of organic carbon in anoxic environments, concurrently with organic matter sulfurization (Raven et al., 2021a). A better understanding of the mechanisms that form associations between organic carbon and iron sulfide minerals is needed. Specifically, it would be critical to determine the type of interactions between Fe and S in iron sulfide minerals and atoms in functional groups of organic carbon (C, H, O, and N), if bonding is covalent or noncovalent, and if organic carbon can be incorporated in the crystal structure of iron sulfide minerals. A better characterization of the organo-mineral interface is required, although technically challenging at high resolution (Kleber et al., 2021). Adsorption and co-precipitation experiments with individual organic molecules originating from biomass, and with a large variety of natural organic matter mixtures will help understand the dynamics of interactions better. While our experiments show stable interactions between iron sulfide minerals and organic carbon in long-term anoxic incubations, it will be necessary to determine the reactivity of these interactions with other microorganisms under anoxic conditions, and their stability under oxic conditions. Iron sulfide minerals store preferentially proteinaceous material, which is the preferred food for some other types of microorganisms in subsurface sediments (Lloyd et al., 2013). It is also unknown if SRM can access this organic carbon for their own metabolism. Iron sulfide minerals could also be an energy source for either sulfide- or iron-oxidizing bacteria. Additionally, the effect of mineral transformations on TC contents needs to be investigated as eventually metastable iron sulfide minerals might transform to pyrite, independently of the pyrite formation pathway, or might be oxidized at the oxic-anoxic interface in sediments. Finally, biogenic FeS minerals could also have an impact on the sequestration of trace metals (Co, Cu, and Ni) and important nutrients (N and P), which would in turn influence microbial life in anoxic environments as well as recorded proxies typically used for the reconstruction of past anoxic environments (Large et al., 2017; Gregory et al., 2019). While marine environments represent the largest environment where microbial sulfate reduction happens and iron sulfide mineral precipitation occurs, it is also important to consider freshwater environments. To conclude, there are still numerous questions to investigate related to interactions between iron sulfide minerals and organic carbon; questions that are relevant for the fields of biogeochemistry of past and modern environments, astrobiology, and microbial physiology.

## Data Availability Statement

The raw data supporting the conclusions of this article will be made available by the authors, without undue reservation.

## Author Contributions

AP conceived and supervised the project, performed the experiments, analyzed the data, and wrote the manuscript. NN and CB performed the experiments, carried out the analyses, and contributed to the writing of the manuscript. All authors contributed to the article and approved the submitted version.

## Funding

This work was funded by a Research Infrastructure grant from the Nevada NASA Space Grant Consortium awarded to AP (grant number NNX15AI02H). NN was supported by a CSUN scholarship from the UNLV Office of Undergraduate Research during the Summer of 2019 and by an NSF EPSCoR Nevada Undergraduate Research Opportunity Program (UROP) scholarship for the academic year 2020-2021. CB was supported by an NSF EPSCoR Nevada undergraduate research opportunity program (UROP) scholarship for the academic year 2021-2022. The publication fees for this article were partially supported by the UNLV University Libraries Open Article Fund.

## Conflict of Interest

The authors declare that the research was conducted in the absence of any commercial or financial relationships that could be construed as a potential conflict of interest.

## Publisher’s Note

All claims expressed in this article are solely those of the authors and do not necessarily represent those of their affiliated organizations, or those of the publisher, the editors and the reviewers. Any product that may be evaluated in this article, or claim that may be made by its manufacturer, is not guaranteed or endorsed by the publisher.
